# Long-term impact of post COVID-19 pandemic quarantine on eating habits changes among adult residents of Riyadh, Saudi Arabia

**DOI:** 10.3389/fnut.2023.1243288

**Published:** 2023-10-05

**Authors:** Mohamad Al-Tannir, Isamme AlFayyad, Mona Altannir, Arwa Alosaimi, Afrah Alonazi, Afnan Alqarni

**Affiliations:** ^1^Research Center, King Fahad Medical City, Riyadh Second Health Cluster, Riyadh, Saudi Arabia; ^2^Nutrition and Dietetics, Eurofins-Ajal, Riyadh, Saudi Arabia; ^3^Family Medicine Department, King Fahad Medical City, Riyadh Second Health Cluster, Riyadh, Saudi Arabia

**Keywords:** COVID-19 pandemic, dietary changes, healthy/unhealthy, Saudi Arabia, impact, long-term

## Abstract

**Background:**

COVID-19 outbreak and quarantine measures clearly had an impact on the population’s eating habits-related behavior.

**Objective:**

This study aimed to explore the long-term impact of the COVID-19 pandemic and physical quarantine on eating habits after quarantine among Riyadh city residents, Saudi Arabia.

**Methods:**

A cross-sectional study was conducted through an online survey between December 2022 and April 2023 on a convenient sample of Saudi adults in Riyadh, Saudi Arabia. A valid questionnaire was used to measure study outcomes. A comparison between dietary habits before and after COVID-19 was performed to identify the changes in dietary habits.

**Results:**

1,451 Saudi adults residing in Riyadh completed the online survey. The majority (88.6%) of the respondents reported changes in dietary habits after COVID-19. About 50% had 1–3 dietary habits changes and slightly more than one-third had 4–7 dietary habits change. About 33.8% of the participants reported stable weight during the COVID-19 pandemic. However, 40.9% reported weight gain, and 20.7% reported weight loss. The participants reported several unhealthy dietary changes most commonly eating fast food (33%), eating junk food/fast food due to boredom/distress/disappointment (29.8%), and high sugar such as sweet porridge, pastry, sweets, and chocolate (28.5%). On the other side, the participants showed healthy dietary habits such as having a balanced healthy ingredients diet (34.4%), an increase in the consumption of fruits and vegetables, and a decrease in the intake of junk foods (28.9%). Males were more likely to exhibit unhealthy dietary habits than females (Odd Ratio:1.43, *p* = 0.038, CI: 1.02–2.02). Increasing age was associated with a reduction in the likelihood of exhibiting unhealthy dietary habits (OR: 0.98, *p* = 0.011, CI: 0.96–0.99). Moreover, participants who reported stable weight or weight loss during COVID-19 were 0.29 (*p* = 0.043, 0.09–0.96) and 0.34 (*p* = 0.020, 0.07–0.79), respectively, less likely to have unhealthy dietary habits.

**Conclusion:**

Although healthy dietary habits have been reported in this study, such as consumption of fruits and vegetables, COVID-19 confinement has also led to negative dietary behaviors reflected by high consumption of fast/junk food and sugar intake resulting in weight gain, a potential adverse impact on the population wellbeing.

## Introduction

The World Health Organization (WHO) declared the coronavirus (COVID-19), a serious acute respiratory condition, to be a pandemic and a public health threat in March 2020 (1). Saudi Arabia announced the first COVID-19 case on March 2, 2020 (2). In step with international efforts to counter and mitigate the spread of COVID-19, Saudi Arabia’s Ministry of Health has applied several preventive measures and restrictions to limit the spread of infection including physical distancing. Physical distancing was implemented in various ways including quarantines, travel restrictions, distance work, and closing of stores, crowded areas including universities, schools, and gym/sport centers (3, 4). Individuals were informed by law of physical distancing methods by staying at home, limiting travel, avoiding overcrowded spots, using non-contact greetings, and physically distancing themselves from others (3, 5). Accordingly, changes in health and socio-economic status have been reported locally and globally (6).

As a result of preventive restrictions, new habits and routines have been developed that need attention (6). A number of studies have shown positive modifications in persons’ lifestyle such as eating habits, and increasing the number of consumed and cooked meals at home (6–8). On the other hand, a home quarantine and limited practice of normal activities had a negative impact on the individual daily habits, such as increased food consumption “emotional eating” to deal with emotional triggers as many persons’ experience undesirable feelings and distress due to fear of infection and of the loss of loved one (6, 7). Additionally, working from home leads to less physical activity (9). An aggravated sedentary lifestyle, increased sitting time, fewer chances for performing physical activity, and extended time spent on smart devices have adversely affected people’s sleep quality (8–14).

By the end of 2021, normal life activities returned gradually particularly for people who completed the doses of COVID-19 vaccine. Several studies have assessed and reported the short-term effect of home isolation and lockdown (8–12). Nevertheless, few studies have evaluated the long-term impact of quarantine including post pandemic effect. However, there is a gap in knowledge on the long-term impact of quarantine on dietary habits from Saudi Arabia and regional countries. It is probable that the pandemic left a heritage of doubt and psychological disturbance among people. It is imperative to investigate whether these alterations in physical activity, eating habits, and sleep quality have a significant impact on people’s daily quality of life (15). Therefore, this study aims to explore the long-term impact of COVID-19 pandemic and physical quarantine on eating habits after quarantine among Saudi adults in Riyadh City, Saudi Arabia.

## Materials and methods

### Study design and setting

An analytical observational cross-sectional study was conducted among a sample of Riyadh City Saudi adults between December 2022 and April 2023.

### Study sample and sampling technique

This study included the Saudi population aged >18 years, were residents in Riyadh city during the quarantine, have no mental disorders or physical disabilities, and were willing to participate in this online survey. A convenient sampling technique was used to recruit study participants. The total population of Saudi Arabia is 32.12 million, and adult Saudis represent about 68% of the total population. The potential participants were identified by three study coordinators for eligibility and shared with the survey via email or WhatsApp upon fulfilling the inclusion criteria. The survey was designed in compliance with the CHERRIES guidelines for web survey (16).

### Data collection procedure

The data collection tool is composed of two sections parts. Section 1 included sociodemographic and anthropometric parameters such as age, sex, height, and weight. The second section assessed the dietary habits changes before and after the COVID-19 pandemic using a previously validated questionnaire (17). The questionnaire was originally written in English and underwent linguistic validation using a forward-backward translation technique by expert translators, and was reconciled by an expert panel. The questionnaire was administered to the participants in the Arabic language. A face validity testing was also conducted by expert researchers and medical physicians to assess the comprehensiveness of the designed questions, and the clarity of wording. Following the face validity testing, a pilot study was performed on 30 parents to compute the reliability of the questionnaire. A Cronbach alpha test showed a score of 0.83 suggesting a good internal consistency. The questionnaire was composed of 12 questions and was completed twice at the same time point. The questionnaire addressed questions about the frequency of maintaining a regular meal pattern, consumption of fast food, fried food, junk food, fruits, and vegetable intake, having a balanced diet with healthy ingredients, intake of milk and its products, pulses, eggs, or meat. In addition, the questionnaire included questions about the frequency of teaspoons consumption of sugar/honey/jiggery, sugar-sweetened beverages, foods with high sugar, and lastly about how often frequency of eating junk food/fast food is due to boredom/distress/disappointment. Questions 1–8 and 10–12 were measured and coded as follows: (Not routinely: 1; 1–2 times/week: 2; 3–4 times/week: 3; 5–6 times/week: 4; and almost daily: 5). For question 9, it was measured as follow: (Zero teaspoons/day, I do not add sugar in my meals/ beverages: 1; 1–2 teaspoons/day: 2; 3–4 teaspoons/day: 3; 5–6 teaspoons/day: 4, and > 6 teaspoons/day: 5).

### Data analysis

We used the SPSS version 22.0 (IBM Corporation, Armonk, NY, United States) 22 to analyze the data. Determination of whether the participants have changes in dietary habits (healthy or unhealthy) was done by deducting the post COVID-19 reported score from the pre COVID-19 reported score. For questions (unhealthy habits) 2–4 and 9–12, if the score is positive [possible range 1–4] then the participants have unhealthy dietary changes, and if the score is negative (worse dietary habit), then the participants have healthy dietary changes. For questions (healthy habits) 1 and 5–8, if the score is negative, then the participants have unhealthy dietary changes, and if the score is positive, then the participants have healthy dietary changes. The number of changes in dietary habits was classified into three categories (1–3 habits, 4–7 habits, and 8–11 habits). The study data were normally distributed. Categorical variables were summarized using descriptive statistics as frequencies, percentages, and continuous variables as means and standard deviation. The chi-square test was used to compare categorical variables. An independent sample *t*-test was used to compare two means, and a one-way ANOVA between the means of two or more continuous variables. The change in dietary habits was transformed into a binary variable (yes/no). We carried out a binary logistic regression to determine the predictors of change in dietary habits. The variables included in the binary logistic regression model were selected based on statistical significance variables with values of *p* < 0.025 on the univariable analysis were included in the model. *p* values of less than 0.05 were considered significant.

### Sample size calculation

The online Epi Info sample size calculator was used to calculate the sample size based on a previous similar study conducted in Saudi Arabia (18) and the Saudi General Authority for Statistics in 2021 (19). The anticipated non-response rate was 20%, with a 99.99% confidence level, a 5% margin error, and a design effect of 1,453 participants required.

## Results

A total of 1,451 participants completed the survey. Males represented 47.6% of the respondents with statistically significant higher age (35.59 ± 13.12, *p* = 0.011), height (172.22 ± 7.54, *p* < 0.001), and weight (65.59 ± 11.45, *p* < 0.001) than females. About one-third of the participants (33.8%) reported stable weight during COVID-19 pandemic. However, 40.9% reported weight gain, and 20.7% reported weight loss. A total of 1,286 (88.6%) indicated changes in their dietary habits after COVID-19 pandemic. Half of them (50.4%) had 1–3 dietary habits change and slightly more than one-third (34.4%) had 4–7 dietary habits change. Further details are presented in Table 1.

**Table 1:** Demographic characteristics of participants and frequency of dietary habits change after COVID-19 pandemic.

Characteristics	*n* (%)	Male (*n* = 690)	Female (*n* = 761)	*p* value
Age (mean ± SD)	34.72 ± 12.38	35.59 ± 13.12	33.93 ± 11.63	**0.011**
Marital status				
Single	579 (39.9)	289 (41.9)	290 (38.1)	0.231
Married	726 (50)	329 (47.7)	397 (52.2)	
Divorced/widowed	146 (10.1)	72 (10.4)	74 (9.7)	
Height (cm)	165.52 ± 9.336	172.22 ± 7.54	159.45	**<0.001**
Weight (Kg)	69.78 ± 12.34	74.41 ± 11.62	65.59 ± 11.45	**<0.001**
Body mass index (mean ± SD)	25.47 ± 4.10	25.07 ± 3.59	25.83 ± 4.45	**<0.001**
Underweight (>18.5)	22 (1.5)	18 (2.6)	4 (0.5)	
Healthy weight (18.5–24.9)	728 (50.2)	349 (50.6)	379 (49.8)	
Overweight (25.0–29.9)	507 (34.9)	257 (37.2)	250 (32.9)	
Obese (≥30)	194 (13.4)	66 (9.6)	128 (16.8)	
Weight during COVID-19 pandemic				0.115
Was stable	491 (33.8)	236 (34.2)	255 (33.5)	
Lost weight	301 (20.7)	130 (18.8)	171 (22.5)	
Gained weight	593 (40.9)	285 (41.3)	308 (40.5)	
I do not know	66 (4.5)	39 (5.7)	27 (3.5)	
Change in dietary habits (mean ± SD)	3.09 ± 2.26	3.26 ± 2.12	2.95 ± 2.29	**0.009**
No	165 (11.4)	67 (9.7)	96 (12.9)	
Yes	1,286 (88.6)	623 (90.3)	663 (87.1)	
*1–3 habits*	732 (50.4)	329 (47.7)	403 (53)	
*4–7 habits*	499 (34.4)	272 (39.4)	227 (29.8)	
*8–11 habits*	55 (3.8)	22 (3.2)	33 (4.3)	

Bold value of *p*: statistically significant at < 0.05; (*n* = 1,451).

Table 2 displays the percentages of dietary habits change before and after COVID-19. The results showed statistically significant difference in the consumption (before and after COVID-19 pandemic) of fast food (worse consumption; *p* < 0.001), fried food (worse consumption; *p* < 0.001), fruits and vegetables (better consumption; *p* < 0.001), balanced healthy ingredients diet (better consumption; *p* < 0.001), sugar/honey/jiggery (*p* < 0.001), sugar-sweetened beverages (worse consumption; *p* < 0.001), and junk food/fast food due to boredom/distress/disappointment (worse consumption; *p* = 0.003). No other significant differences were found in the remaining dietary habits (Table 2).

**Table 2:** Comparison of dietary habits change before and after COVID-19.

Dietary habits	Before COVID-19	After COVID-19	*p* value
1. *How often do you maintain a regular meal pattern?*			0.750
Not routinely	462 (31.8)	446 (30.7)	0.643
1–2 times/week	215 (14.8)	237 (16.3)	0.335
3–4 times/week	460 (31.7)	454 (31.3)	0.862
5–6 times/week	167 (11.5)	176 (12.1)	0.645
Almost daily	147 (10.1)	138 (9.5)	0.610
2. *How often do you consume fast food such as pizza, burger, pasta or noodles as snacks or meals?*			**<0.001**
Not routinely	415 (28.6)	371 (25.6)	0.163
1–2 times/week	543 (37.4)	486 (33.5)	0.126
3–4 times/week	347 (23.9)	409 (28.2)	**0.044**
5–6 times/week	112 (7.7)	124 (8.5)	0.452
Almost daily	34 (2.3)	61 (5.2)	**0.006**
3. *How often do you consume fried food (fried bread/poori, fried snack such as fries)?*			**<0.001**
Not routinely	530 (36.5)	541 (37.3)	0.751
1–2 times/week	450 (31.0)	441 (30.4)	0.812
3–4 times/week	358 (24.7)	296 (20.4)	**0.030**
5–6 times/week	86 (5.9)	119 (8.2)	**0.024**
Almost daily	27 (1.9)	54 (3.7)	**0.029**
4. *How often do you consume junk foods (popcorn, chips, candies, and chocolate) as snacks?*			0.641
Not routinely	367 (25.3)	397 (27.5)	0.313
1–2 times/week	367 (25.3)	370 (25.6)	0.888
3–4 times/week	452 (31.2)	419 (29.0)	0.349
5–6 times/week	150 (15.3)	145 (10.1)	0.802
Almost daily	115 (7.9)	114 (7.9)	0.969
5. *What was the frequency of your fruits and vegetables intake?*			**0.010**
Not routinely	334 (23.0)	279 (19.2)	**0.043**
1–2 times/week	334 (23.4)	336 (23.2)	0.944
3–4 times/week	479 (33.0)	478 (32.9)	0977
5–6 times/week	178 (12.3)	182 (12.5)	0.842
Almost daily	126 (8.7)	176 (12.1)	**0.006**
6. *How often do you have a balanced diet by including healthy ingredients (whole wheat, pulses, legumes, eggs, and nuts) in your meals?*			**<0.001**
Not routinely	331 (22.8)	241 (16.6)	**<0.001**
1–2 times/week	289 (19.9)	288 (19.8)	0.969
3–4 times/week	530 (36.5)	557 (38.4)	0.484
5–6 times/week	190 (13.1)	214 (14.7)	0.263
Almost daily	111 (7.6)	151 (10.4)	**0.017**
7. *How often do you have 2–3 servings of milk or its products (curd, buttermilk, cheese, paneer etc.) in a day?*			0.769
Not routinely	228 (15.7)	207 (14.3)	0.347
1–2 times/week	309 (21.3)	316 (21.8)	0.799
3–4 times/week	494 (34.0)	506 (34.9)	0.743
5–6 times/week	221 (15.2)	211 (14.5)	0.653
Almost daily	199 (13.7)	211 (14.5)	0.579
8. *How often do you have one or more servings of pulses, egg or meat in a day?*			
Not routinely	137 (9.4)	129 (8.9)	0.173
1–2 times/week	206 (14.2)	212 (14.6)	0.638
3–4 times/week	432 (29.8)	377 (26.0)	0.983
5–6 times/week	254 (17.5)	275 (19.0)	0.087
Almost daily	422 (29.1)	458 (31.6)	0.401
9. *How many teaspoons of sugar/honey/jiggery do you consume in a day?*			**<0.001**
Zero teaspoons/day, I don’ t add sugar in my meals/beverages	270 (18.6)	411 (28.3)	**<0.001**
1–2 teaspoons/day	611 (42.1)	536 (36.9)	0.060
3–4 teaspoons/day	406 (28.0)	330 (22.7)	**0.012**
5–6 teaspoons/day	128 (8.8)	124 (8.5)	0.809
More than 6 teaspoons/day	36 (2.5)	50 (3.4)	0.136
10. *How often do you consume sugar-sweetened beverages (juice, soft drinks, and flavored soda)?*			**<0.001**
Not routinely	461 (31.8)	531 (36.6)	0.054
1–2 times/week	326 (22.5)	313 (21.6)	0.641
3–4 times/week	427 (29.4)	336 (23.2)	**0.003**
5–6 times/week	119 (8.2)	126 (8.7)	0.667
Almost daily	118 (8.2)	145 (10.0)	0.110
11. *How often do you consume foods with high sugar such sweet porridge, pastry, sweets and chocolate etc.?*			**0.062**
Not routinely	524 (36.1)	553 (38.1)	0.450
1–2 times/week	401 (27.6)	352 (24.3)	0.111
3–4 times/week	358 (24.7)	365 (25.1)	0.840
5–6 times/week	114 (7.9)	103 (7.1)	0.471
Almost daily	54 (3.7)	78 (5.4)	**0.041**
12. *How often do you eat junk food/fast food due to boredom/distress/disappointment?*			
Not routinely	630 (43.4)	615 (42.4)	**0.003**
1–2 times/week	396 (27.3)	348 (24.0)	0.722
3–4 times/week	325 (22.4)	333 (22.9)	0.116
5–6 times/week	51 (3.5)	86 (5.9)	0.778
Almost daily	49 (3.4)	69 (4.8)	**0.003**

Bold *p* values: statistically significant at < 0.05; (*n* = 1,451).

The participants reported several unhealthy dietary changes most commonly eating fast food (33%), eating junk food/fast food due to boredom/distress/disappointment (29.8%), and high sugar such as sweet porridge, pastry, sweets, and chocolate (28.5%). In contrast, the top healthier reported dietary habits changes were having a balanced healthy ingredients diet (34.4%), an increase in the consumption of fruits and vegetables, and a decrease in the intake of junk foods (28.9%: Table 3).

**Table 3:** Changes in dietary habits.

Questions	*n* (%)
■ How often do you maintain a regular meal pattern?	
*No change*	674 (46.5)
*Unhealthy change*	390 (26.9)
*Healthy change*	387 (26.6)
■ How often do you consume fast food such as pizza, burger, pasta, or noodles as snacks or meals?	
*No change*	642 (44.2)
*Unhealthy change*	**478 (33.0)** ^*^
*Healthy change*	331 (22.8)
■ How often do you consume fried food (fried bread/poori, fried snack such as fries)?	
*No change*	689 (47.5)
*Unhealthy change*	404 (27.8)
*Healthy change*	358 (24.7)
■ How often do you consume junk foods (popcorn, chips, candies, and chocolate) as snacks?	
*No change*	654 (45.1)
*Unhealthy Change*	377 (26.0)
*Healthy Change*	**420 (28.9)** ^**¥**^
■ What was the frequency of your fruits and vegetables intake?	
*No change*	670 (46.2)
*Unhealthy Change*	340 (23.4)
*Healthy Change*	**441 (30.4)** ^**¥**^
■ How often do you have a balanced diet by including healthy ingredients (whole wheat, pulses, legumes, eggs, nuts,) in your meals?	
*No change*	630 (43.4)
*Unhealthy change*	322 (22.2)
*Healthy change*	**499 (34.4)** ^**¥**^
■ How often do you have 2–3 servings of milk or its products (curd, buttermilk, cheese, paneer etc.) in a day?	
*No change*	652 (45.0)
*Unhealthy change*	392 (27.0)
*Healthy change*	407 (28.0)
■ How often do you have one or more servings of pulses, egg or meat in a day?	
*No change*	770 (53.1)
*Unhealthy Change*	305 (21.0)
*Healthy Change*	376 (25.9)
■ How many teaspoons of sugar/honey/jiggery do you consume in a day?	
*No change*	791 (54.5)
*Unhealthy change*	258 (17.8)
*Healthy change*	402 (27.7)
■ How often do you consume sugar-sweetened beverages (juice, soft drinks, flavored soda)?	
*No change*	647 (46.5)
*Unhealthy change*	377 (26.0)
*Healthy change*	400 (27.5)
■ How often do you consume foods with high sugar such sweet porridge, pastry, sweets and chocolate etc.?	
*No change*	659 (45.4)
*Unhealthy change*	**414 (28.5)** ^*^
*Healthy change*	378 (26.1)
■ How often do you eat junk food/fast food due to boredom/distress/disappointment?	
*No change*	709 (48.9)
*Unhealthy change*	**433 (29.8)** ^*^
*Healthy change*	309 (21.3)

^*^Top 3 unhealthy dietary habit changes; ^¥^Top 3 unhealthy dietary habit changes; (*n* = 1,451). The bold values in table indicate the statistically significant *p* - values at < 0.05.

The univariate analyses showed a significant association between age (*p* = 0.010) and weight status during COVID-19 (*p* < 0.001) with unhealthy dietary habits changes after COVID-19. A logistic regression was performed to ascertain the effects of age, sex, BMI, marital status, and weight during COVID-19 pandemic on the likelihood that participants exhibit unhealthy dietary habits. Males were 1.43 times more likely to exhibit unhealthy dietary habits than females (*p* = 0.038, CI: 1.02–2.02). Increasing age was associated with a reduction in the likelihood of exhibiting unhealthy dietary habits (OR: 0.98, *p* = 0.011, CI: 0.96–0.99). Moreover, participants who reported stable weight or weight loss during COVID-19 were 0.29 (*p* = 0.043, 0.09–0.96) and 0.34 (*p* = 0.020, 0.07–0.79), respectively, less likely to have unhealthy dietary habits (Table 4).

**Table 4:** Predictors of dietary habits changes (*n* = 1,451).

Characteristics	*n* (%) of participants in each dietary habits change category			Multivariate analysis			
	Unhealthy change	Healthy change	*p* value	Unadjusted OR [95% CI]	*p* value	Adjusted OR [95% CI]	*p* value
Age	34.42 ± 12.35	37.07 ± 12.48	**0.010**	0.98 [0.97–0.99]	0.010	098 [0.96–0.99]	**0.011**
Sex							
*Male*	623 (48.4)	67 (40.6)	0.058	1.37 [0.99–1.91]	0.058	1.43 [0.97–1.07]	**0.038**
Female^*^	663 (51.6)	98 (59.8)		-		-	
BMI (Kg/m^2^)	25.51 ± 4.11	25.09 ± 4.03	0.218	1.03 [0.99–1.07]	0.218	1.02 [0.97–1.07]	0.444
Marital status							
*Single*	523 (40.7)	56 (33.9)	0.120	0.99 [0.54–1.83]	0.976	0.71 [0.35–1.42]	0.327
*Married*	631 (49.1)	95 (57.6)		0.70 [0.93–1.27]	0.246	0.69 [0.38–1.27]	0.241
Divorced/Widow^*^	132 (10.3)	14 (8.5)		-		-	
Weight during COVID-19 pandemic							
*Was stable*	419 (32.6)	72 (43.6)	**<0.001**	0.28 [0.09–0.91]	0.034	0.29 [0.09–0.96]	**0.043**
*Lost weight*	250 (19.4)	51 (30.9)		0.23 [0.07–0.77]	0.017	0.34 [0.07–0.79]	**0.020**
*Gained weight*	554 (43.1)	39 (23.6)		0.68 [0.20–2.25]	0.424	0.68 [0.21–2.27]	0.524
I do not know^*^	63 (4.9)	3 (1.8)		-		-	-

^*^Reference variable; (*n* = 1,451). The bold values in table indicate the statistically significant *p* - values at < 0.05.

## Discussion

COVID-19 outbreak and quarantine measures clearly had an impact on the population’s lifestyle-related behavior. To control the outbreak of COVID-19, many countries, including Saudi Arabia, have implemented quarantine measures. The quarantine had the beneficial results of lowering the pandemic level due to the measures implemented. However, the fear of illness and death as well as quarantine measures boosted people’s stress levels and made them change their regular behaviors. Our study evaluated the long-term impact of the COVID-19 quarantine on the eating habits of adults from Riyadh, Saudi Arabia.

The results from the current study showed that 40.9% of participants reported weight gain during the COVID-19 quarantine. Our results are in line with previous studies that have reported weight gain during lockdown periods in Saudi Arabia (10, 18, 20–22). These studies have reported a prevalence of weight gain between 29.1 and 62.3%. Our findings are consistent as well with previous findings from different regions worldwide. A recent meta-analysis published by Anderson et al. (23) showed a small but potentially clinically significant increase in weight gain, BMI, and prevalence of obesity in both children and adults during COVID-19. Another systematic review and meta-analysis on the effects of COVID-19 lockdown on eating disorders and obesity reported 52% pooled prevalence of increased weight (24). Additionally, a combined systematic review and a meta-analysis have reported a prevalence change in body weight of 11.1–72.4% during COVID-19 lockdown (25). People often overstocked their kitchens with different foods out of fear of COVID-19, which may have resulted in overeating, particularly canned foods, which are high in calories. Given that many people stopped their regular daily routine activities, gyms were closed, and they were forced to work from home, weight gain is a reasonable result of this drop in physical activity and energy expenditure (10, 26, 27). Overall, 80.6% of the participants reported changes in dietary habits due to COVID-19 pandemic.

We found a statistically significant increment in healthy eating habits related to fruits and vegetables and healthy ingredients diet before and after COVID-19 pandemic. This is in accordance with studies of surveyed individuals who have increased their intake of fruits and vegetables (10, 22, 28, 29), and healthy ingredients diet (17, 30). Nonetheless, other studies have reported a decrease in the consumption of fruits and vegetables and healthy ingredients diet. Lippi et al. (31) concluded there is a reduction in fruits and vegetables intake. Similarly, a study in Zimbabwe showed that 57.8% of participants indicated a decrease in the consumption of fruits and vegetables (32). On the other side, we also observed a statistically significant increment in the consumption of fast food, fried food, sugar, consume sugar-sweetened beverages. In previous studies evaluating different perspectives on eating, the participants reported increased consumption of such unhealthy foods (13, 33–37). Conversely, few studies reported a downward trend consumption of fast food during the lockdown restrictions (22, 24, 25, 38).

Boredom has been associated with unhealthy eating behaviors such as higher fat, carbohydrate, and weight gain (39). Staying home for long periods and the high prevalence of sleep disorders during COVID-19 might increase the feeling of boredom, which is often linked with overeating to escape boredom (40). Our results were consistent with the aforementioned where around 30% have eaten junk food/fast food due to boredom/distress/disappointment.

We found that high age, being male, and maintaining or losing weight were significantly associated with reporting healthy dietary behaviors. Younger adults were more likely to have undesired changes in healthy dietary behaviors as consumption of fast food compared with old adults (41). A previous study has reported that females reported unhealthier dietary behaviors and weight gain during COVID-19 pandemic (29).

Nevertheless, our study findings imply that the COVID-19 pandemic and associated regulations have a significant impact on people’s eating habits and food consumption patterns. Although several health recommendations were publicized for healthy eating during the COVID-19 pandemic and distributed information on how an adequate diet can support the immune system, the majority of our study participants did not adopt these recommendations into practice during and after the pandemic.

This work represents one of the few studies on the impact of COVID-19 quarantine on dietary habits changes, some limitations must be recognized. First, this study was cross-sectional, completed at one-time point and the self-reporting by participants on dietary habits changes before COVID-19 can introduce recall bias. The convenient sampling method might have introduced selection bias such that people with negative health and dietary changes were more likely to be interested in and completed the survey and It is still possible that selection bias influenced the results of this study.

## Conclusion

Although healthy dietary habits have been reported in this study, such as the consumption of fruits and vegetables, COVID-19 confinement has also led to negative dietary behaviors reflected by high consumption of fast/junk food and sugar intake resulting in weight gain, a potential adverse impact on the population wellbeing.

## Data availability statement

The raw data supporting the conclusions of this article will be made available by the authors, without undue reservation.

## Ethics statement

The studies involving humans were approved by King Fahad Medical City—institutional review Board. The studies were conducted in accordance with the local legislation and institutional requirements. The ethics committee/institutional review board waived the requirement of written informed consent for participation from the participants or the participants’ legal guardians/next of kin because No risk is anticipated in this survey-based.

## Author contributions

MohA and IA: designed the study, statistical analysis, and supervision. MonA, ArA, AfrA, and AfnA: data collection, literature review, and prepare the draft manuscript. MohA, MonA, and IA: critical revision of the manuscript. All authors contributed to the article and approved the submitted version.
